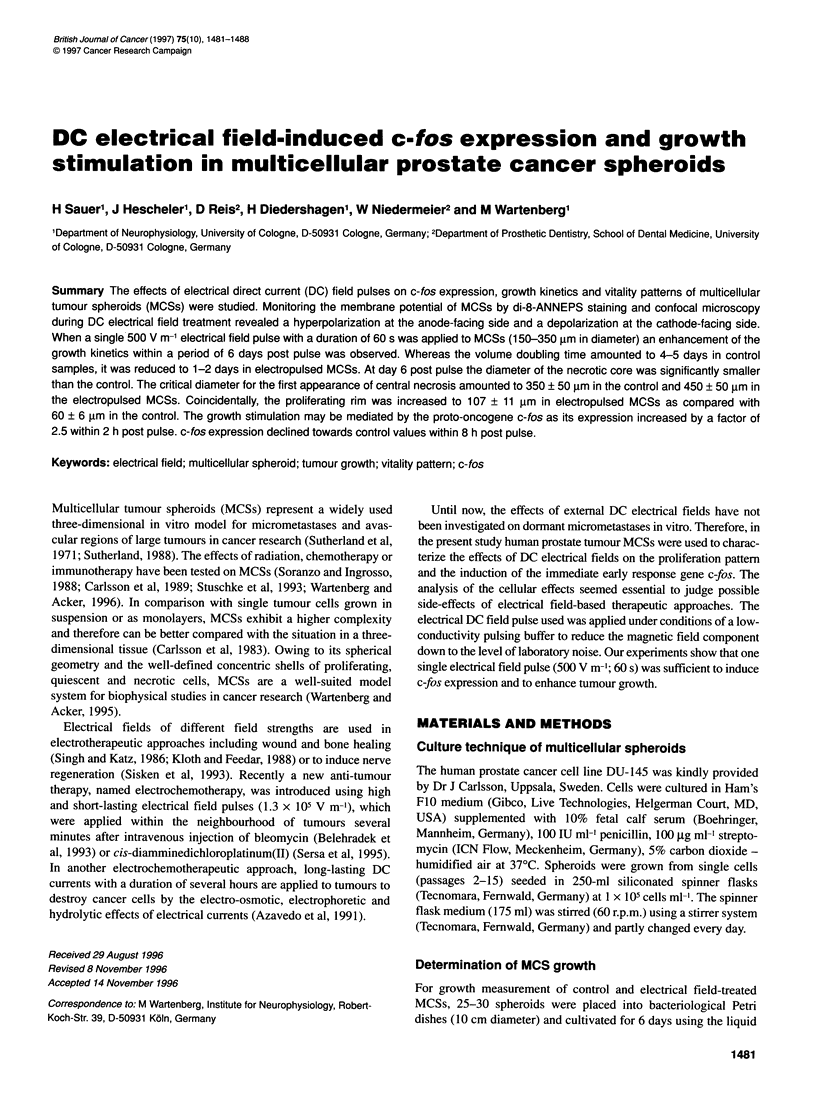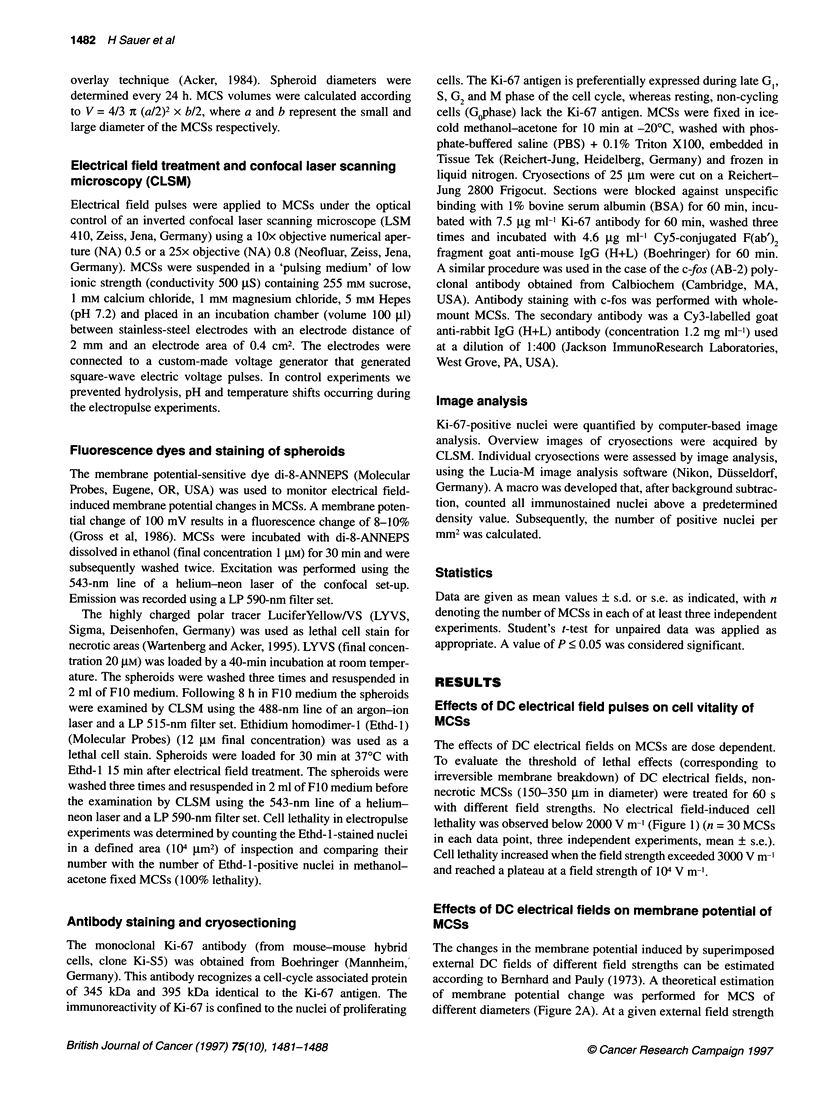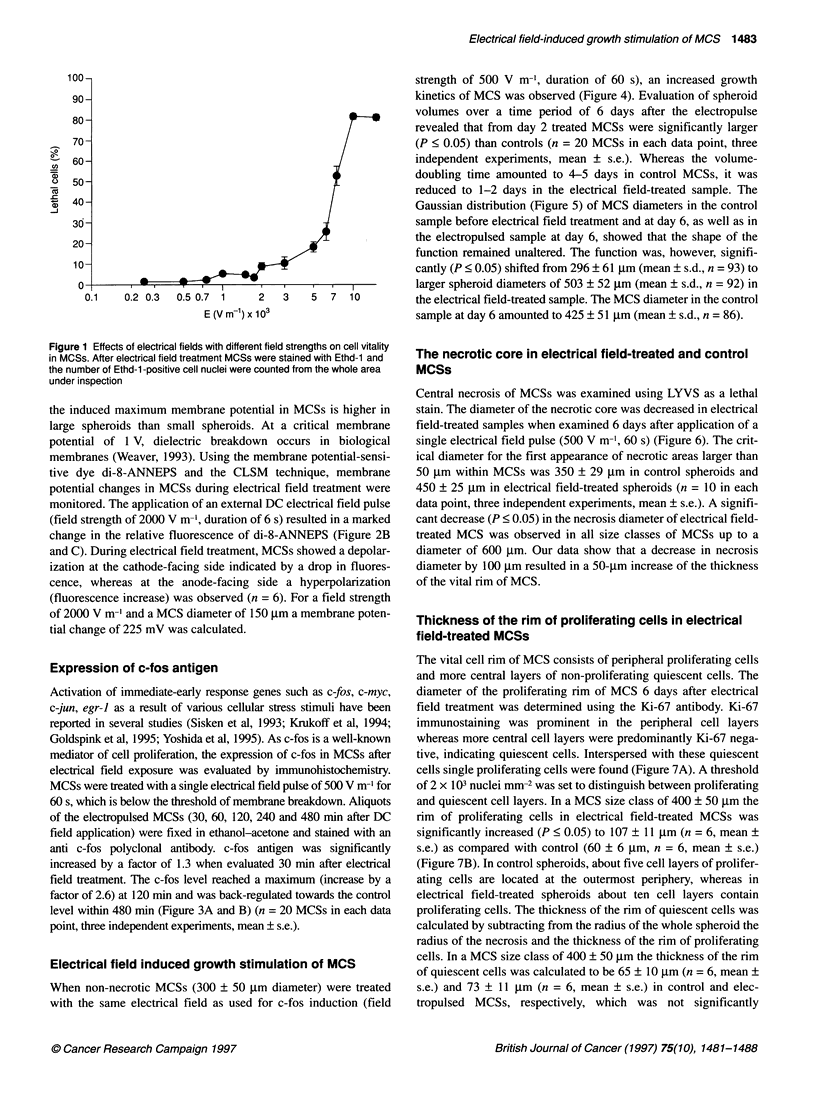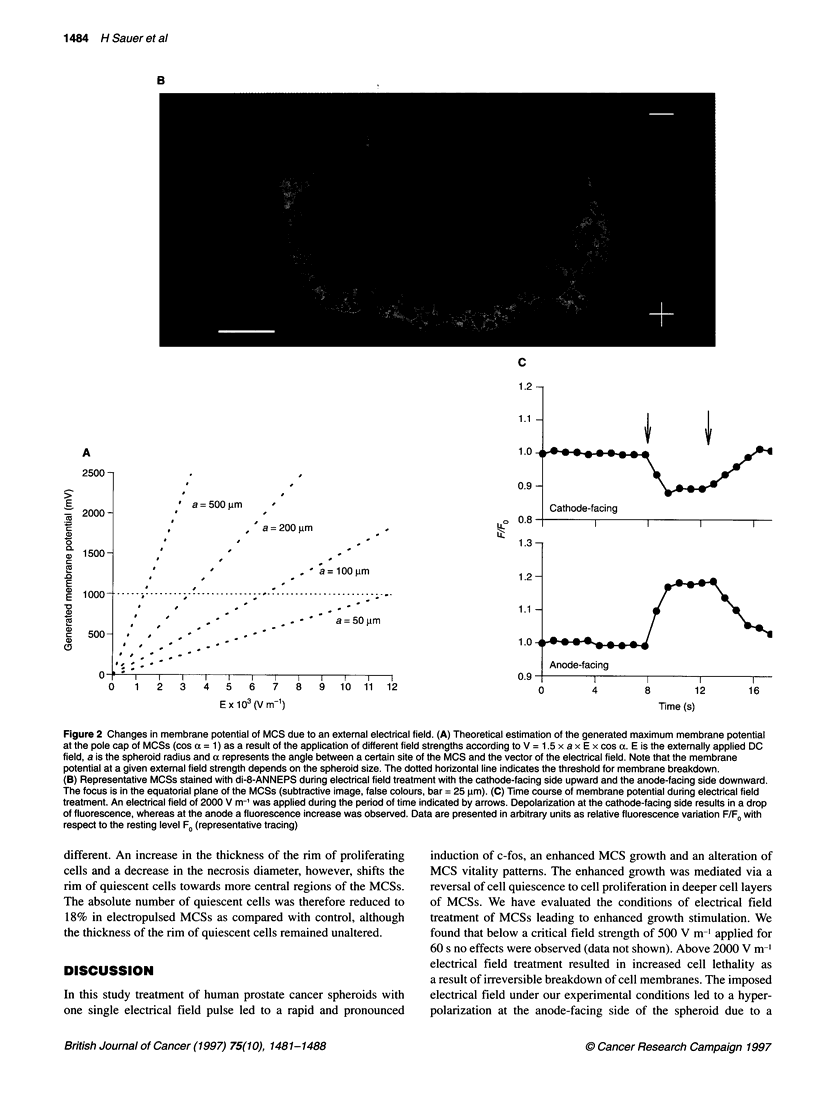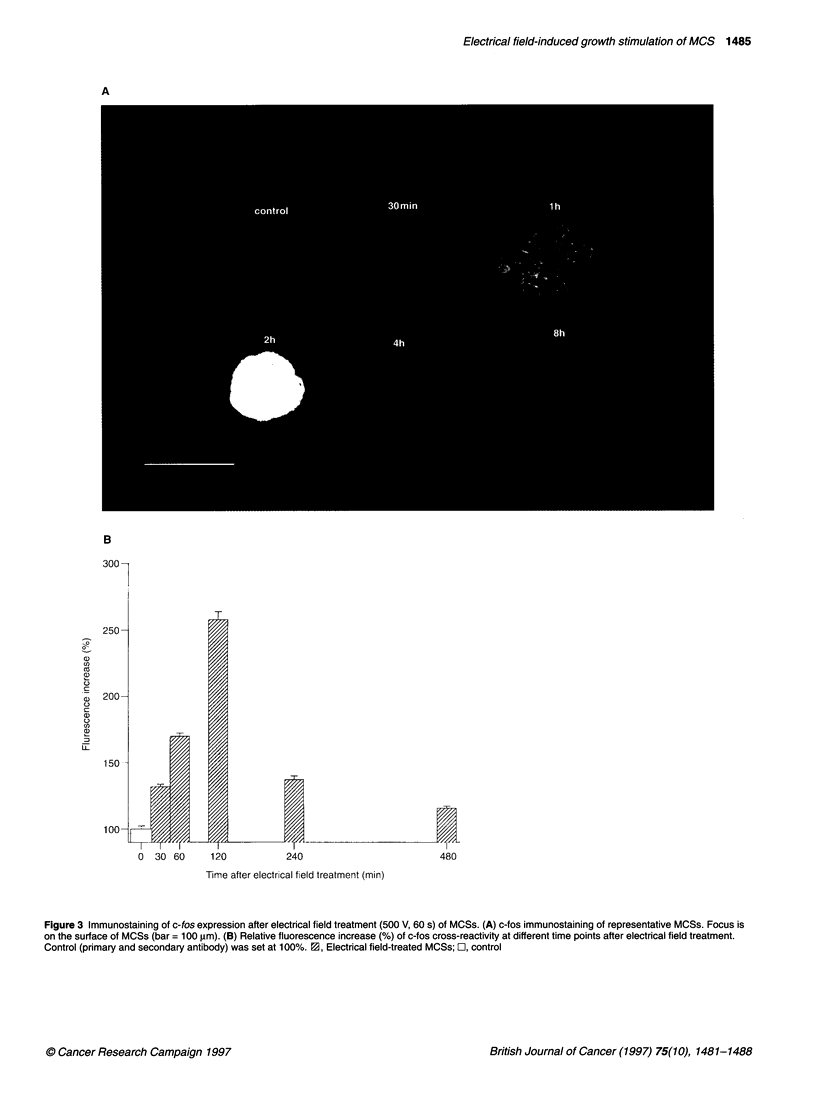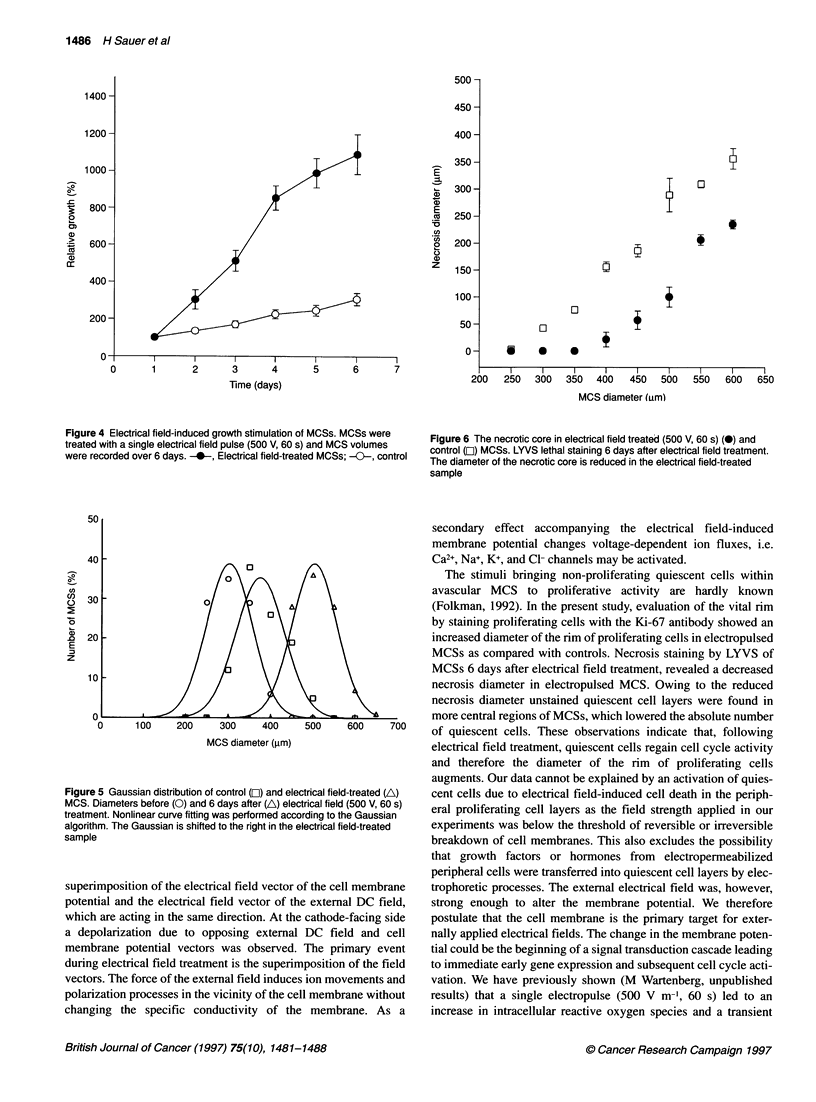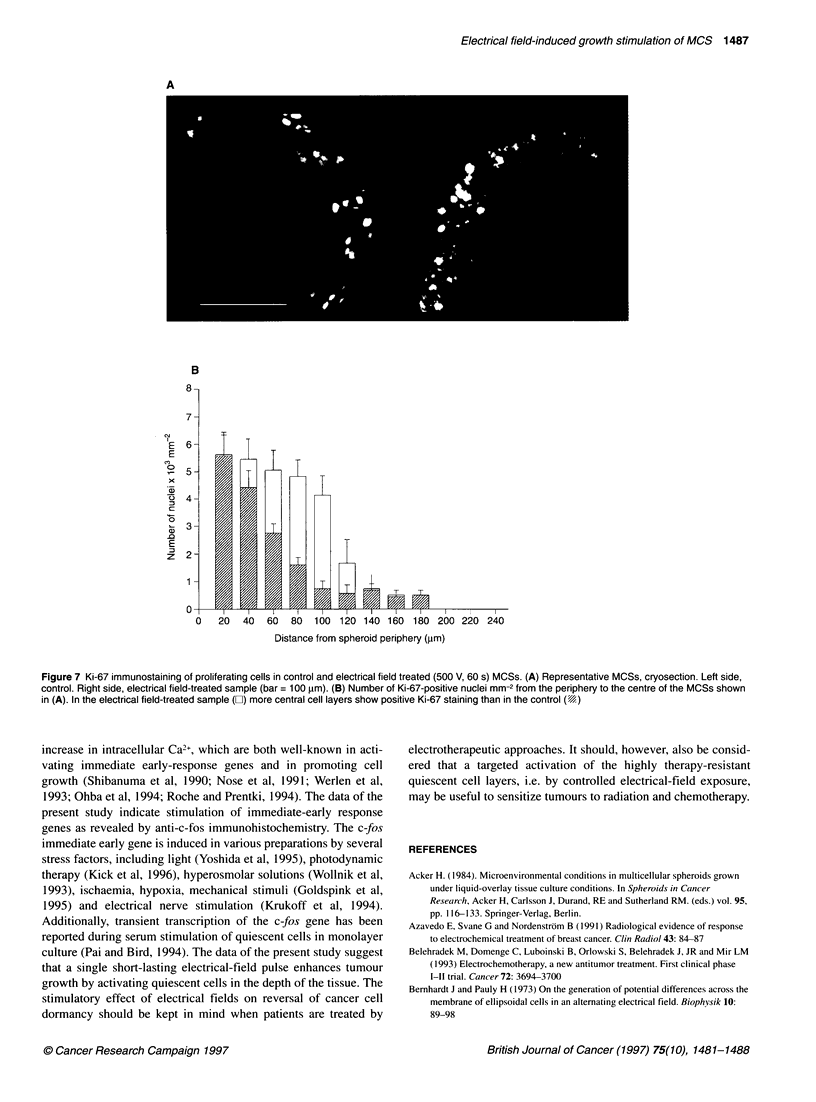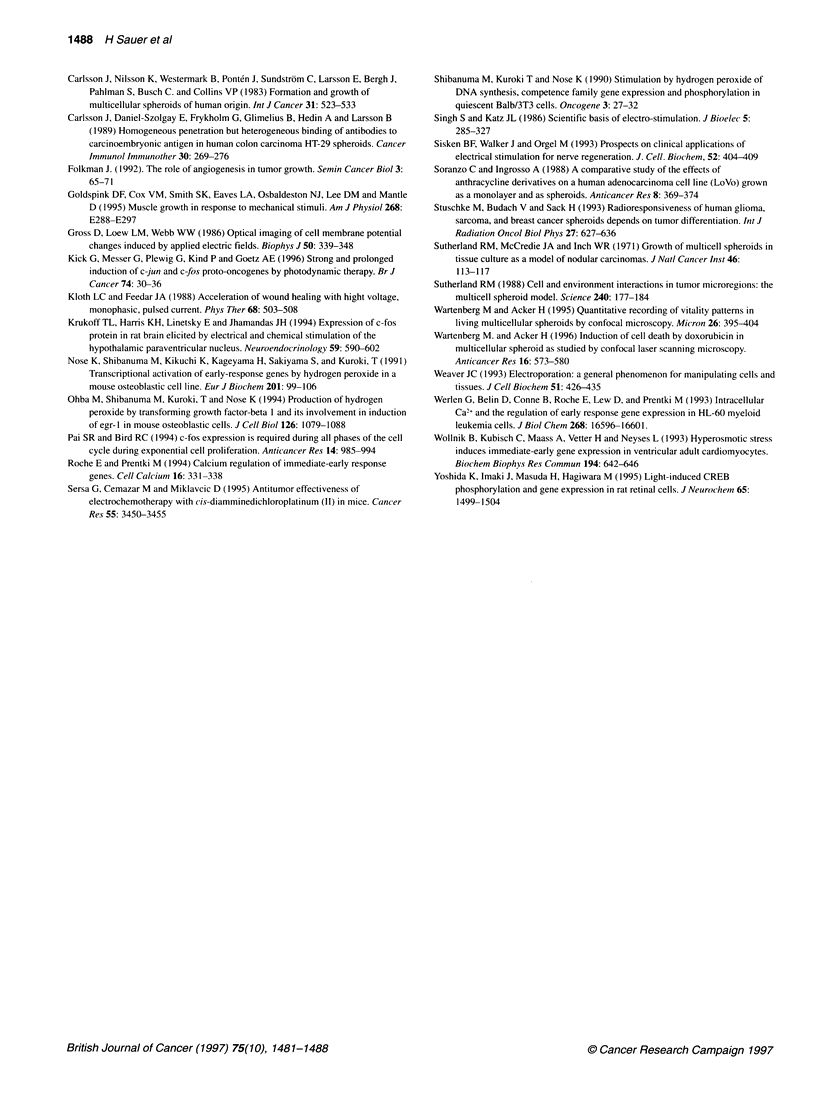# DC electrical field-induced c-fos expression and growth stimulation in multicellular prostate cancer spheroids.

**DOI:** 10.1038/bjc.1997.253

**Published:** 1997

**Authors:** H. Sauer, J. Hescheler, D. Reis, H. Diedershagen, W. Niedermeier, M. Wartenberg

**Affiliations:** Department of Neurophysiology, University of Cologne, Germany.

## Abstract

**Images:**


British Journal of Cancer (1997) 75(10), 1481–1488
© 1997 Cancer Research Campaign

### DC electrical field-induced c-*fos* expression and growth stimulation in multicellular prostate cancer spheroids

**H Sauer[1], J Hescheler[1], D Reis[2], H Diedershagen[1], W Niedermeier[2] and M Wartenberg[1]**

[1]Department of Neurophysiology, University of Cologne, D-50931 Cologne, Germany; [2]Department of Prosthetic Dentistry, School of Dental Medicine, University of Cologne, D-50931 Cologne, Germany

**Summary** The effects of electrical direct current (DC) field pulses on c-*fos* expression, growth kinetics and vitality patterns of multicellular tumour spheroids (MCSs) were studied. Monitoring the membrane potential of MCSs by di-8-ANNEPS staining and confocal microscopy during DC electrical field treatment revealed a hyperpolarization at the anode-facing side and a depolarization at the cathode-facing side. When a single 500 V $m^{-1}$ electrical field pulse with a duration of 60 s was applied to MCSs (150–350 μm in diameter) an enhancement of the growth kinetics within a period of 6 days post pulse was observed. Whereas the volume doubling time amounted to 4–5 days in control samples, it was reduced to 1–2 days in electropulsed MCSs. At day 6 post pulse the diameter of the necrotic core was significantly smaller than the control. The critical diameter for the first appearance of central necrosis amounted to 350 ± 50 μm in the control and 450 ± 50 μm in the electropulsed MCSs. Coincidentally, the proliferating rim was increased to 107 ± 11 μm in electropulsed MCSs as compared with 60 ± 6 μm in the control. The growth stimulation may be mediated by the proto-oncogene c-*fos* as its expression increased by a factor of 2.5 within 2 h post pulse. c-*fos* expression declined towards control values within 8 h post pulse.

**Keywords:** electrical field; multicellular spheroid; tumour growth; vitality pattern; c-*fos*

Multicellular tumour spheroids (MCSs) represent a widely used three-dimensional in vitro model for micrometastases and avascular regions of large tumours in cancer research (Sutherland et al, 1971; Sutherland, 1988). The effects of radiation, chemotherapy or immunotherapy have been tested on MCSs (Soranzo and Ingrosso, 1988; Carlsson et al, 1989; Stuschke et al, 1993; Wartenberg and Acker, 1996). In comparison with single tumour cells grown in suspension or as monolayers, MCSs exhibit a higher complexity and therefore can be better compared with the situation in a three-dimensional tissue (Carlsson et al, 1983). Owing to its spherical geometry and the well-defined concentric shells of proliferating, quiescent and necrotic cells, MCSs are a well-suited model system for biophysical studies in cancer research (Wartenberg and Acker, 1995).

Electrical fields of different field strengths are used in electrotherapeutic approaches including wound and bone healing (Singh and Katz, 1986; Kloth and Feedar, 1988) or to induce nerve regeneration (Sisken et al, 1993). Recently a new anti-tumour therapy, named electrochemotherapy, was introduced using high and short-lasting electrical field pulses ($1.3 \times 10^5$ V $m^{-1}$), which were applied within the neighbourhood of tumours several minutes after intravenous injection of bleomycin (Belehradek et al, 1993) or *cis*-diamminedichloroplatinum(II) (Sersa et al, 1995). In another electrochemotherapeutic approach, long-lasting DC currents with a duration of several hours are applied to tumours to destroy cancer cells by the electro-osmotic, electrophoretic and hydrolytic effects of electrical currents (Azavedo et al, 1991).

*Received 29 August 1996*
*Revised 8 November 1996*
*Accepted 14 November 1996*

*Correspondence to:* M Wartenberg, Institute for Neurophysiology, Robert-Koch-Str. 39, D-50931 Köln, Germany

Until now, the effects of external DC electrical fields have not been investigated on dormant micrometastases in vitro. Therefore, in the present study human prostate tumour MCSs were used to characterize the effects of DC electrical fields on the proliferation pattern and the induction of the immediate early response gene c-*fos*. The analysis of the cellular effects seemed essential to judge possible side-effects of electrical field-based therapeutic approaches. The electrical DC field pulse used was applied under conditions of a low-conductivity pulsing buffer to reduce the magnetic field component down to the level of laboratory noise. Our experiments show that one single electrical field pulse (500 V $m^{-1}$; 60 s) was sufficient to induce c-*fos* expression and to enhance tumour growth.

### MATERIALS AND METHODS

### Culture technique of multicellular spheroids

The human prostate cancer cell line DU-145 was kindly provided by Dr J Carlsson, Uppsala, Sweden. Cells were cultured in Ham's F10 medium (Gibco, Live Technologies, Helgerman Court, MD, USA) supplemented with 10% fetal calf serum (Boehringer, Mannheim, Germany), 100 IU $ml^{-1}$ penicillin, 100 μg $ml^{-1}$ streptomycin (ICN Flow, Meckenheim, Germany), 5% carbon dioxide – humidified air at 37°C. Spheroids were grown from single cells (passages 2–15) seeded in 250-ml siliconated spinner flasks (Tecnomara, Fernwald, Germany) at $1 \times 10^5$ cells $ml^{-1}$. The spinner flask medium (175 ml) was stirred (60 r.p.m.) using a stirrer system (Tecnomara, Fernwald, Germany) and partly changed every day.

### Determination of MCS growth

For growth measurement of control and electrical field-treated MCSs, 25–30 spheroids were placed into bacteriological Petri dishes (10 cm diameter) and cultivated for 6 days using the liquid overlay technique (Acker, 1984). Spheroid diameters were determined every 24 h. MCS volumes were calculated according to $V = 4/3\ \pi\ (a/2)^2 \times b/2$, where $a$ and $b$ represent the small and large diameter of the MCSs respectively.

### Electrical field treatment and confocal laser scanning microscopy (CLSM)

Electrical field pulses were applied to MCSs under the optical control of an inverted confocal laser scanning microscope (LSM 410, Zeiss, Jena, Germany) using a 10× objective numerical aperture (NA) 0.5 or a 25× objective (NA) 0.8 (Neofluar, Zeiss, Jena, Germany). MCSs were suspended in a 'pulsing medium' of low ionic strength (conductivity 500 μS) containing 255 mM sucrose, 1 mM calcium chloride, 1 mM magnesium chloride, 5 mM Hepes (pH 7.2) and placed in an incubation chamber (volume 100 μl) between stainless-steel electrodes with an electrode distance of 2 mm and an electrode area of 0.4 $cm^2$. The electrodes were connected to a custom-made voltage generator that generated square-wave electric voltage pulses. In control experiments we prevented hydrolysis, pH and temperature shifts occurring during the electropulse experiments.

### Fluorescence dyes and staining of spheroids

The membrane potential-sensitive dye di-8-ANNEPS (Molecular Probes, Eugene, OR, USA) was used to monitor electrical field-induced membrane potential changes in MCSs. A membrane potential change of 100 mV results in a fluorescence change of 8–10% (Gross et al, 1986). MCSs were incubated with di-8-ANNEPS dissolved in ethanol (final concentration 1 μM) for 30 min and were subsequently washed twice. Excitation was performed using the 543-nm line of a helium–neon laser of the confocal set-up. Emission was recorded using a LP 590-nm filter set.

The highly charged polar tracer LuciferYellow/VS (LYVS, Sigma, Deisenhofen, Germany) was used as lethal cell stain for necrotic areas (Wartenberg and Acker, 1995). LYVS (final concentration 20 μM) was loaded by a 40-min incubation at room temperature. The spheroids were washed three times and resuspended in 2 ml of F10 medium. Following 8 h in F10 medium the spheroids were examined by CLSM using the 488-nm line of an argon–ion laser and a LP 515-nm filter set. Ethidium homodimer-1 (Ethd-1) (Molecular Probes) (12 μM final concentration) was used as a lethal cell stain. Spheroids were loaded for 30 min at 37°C with Ethd-1 15 min after electrical field treatment. The spheroids were washed three times and resuspended in 2 ml of F10 medium before the examination by CLSM using the 543-nm line of a helium–neon laser and a LP 590-nm filter set. Cell lethality in electropulse experiments was determined by counting the Ethd-1-stained nuclei in a defined area ($10^4$ $\mu m^2$) of inspection and comparing their number with the number of Ethd-1-positive nuclei in methanol–acetone fixed MCSs (100% lethality).

### Antibody staining and cryosectioning

The monoclonal Ki-67 antibody (from mouse–mouse hybrid cells, clone Ki-S5) was obtained from Boehringer (Mannheim, Germany). This antibody recognizes a cell-cycle associated protein of 345 kDa and 395 kDa identical to the Ki-67 antigen. The immunoreactivity of Ki-67 is confined to the nuclei of proliferating cells. The Ki-67 antigen is preferentially expressed during late $G_1$, S, $G_2$ and M phase of the cell cycle, whereas resting, non-cycling cells ($G_0$phase) lack the Ki-67 antigen. MCSs were fixed in ice-cold methanol–acetone for 10 min at –20°C, washed with phosphate-buffered saline (PBS) + 0.1% Triton X100, embedded in Tissue Tek (Reichert-Jung, Heidelberg, Germany) and frozen in liquid nitrogen. Cryosections of 25 μm were cut on a Reichert–Jung 2800 Frigocut. Sections were blocked against unspecific binding with 1% bovine serum albumin (BSA) for 60 min, incubated with 7.5 μg $ml^{-1}$ Ki-67 antibody for 60 min, washed three times and incubated with 4.6 μg $ml^{-1}$ Cy5-conjugated $F(ab')_2$ fragment goat anti-mouse IgG (H+L) (Boehringer) for 60 min. A similar procedure was used in the case of the c-*fos* (AB-2) polyclonal antibody obtained from Calbiochem (Cambridge, MA, USA). Antibody staining with c-fos was performed with whole-mount MCSs. The secondary antibody was a Cy3-labelled goat anti-rabbit IgG (H+L) antibody (concentration 1.2 mg $ml^{-1}$) used at a dilution of 1:400 (Jackson ImmunoResearch Laboratories, West Grove, PA, USA).

### Image analysis

Ki-67-positive nuclei were quantified by computer-based image analysis. Overview images of cryosections were acquired by CLSM. Individual cryosections were assessed by image analysis, using the Lucia-M image analysis software (Nikon, Düsseldorf, Germany). A macro was developed that, after background subtraction, counted all immunostained nuclei above a predetermined density value. Subsequently, the number of positive nuclei per $mm^2$ was calculated.

### Statistics

Data are given as mean values ± s.d. or s.e. as indicated, with $n$ denoting the number of MCSs in each of at least three independent experiments. Student's $t$-test for unpaired data was applied as appropriate. A value of $P \leq 0.05$ was considered significant.

### RESULTS

### Effects of DC electrical field pulses on cell vitality of MCSs

The effects of DC electrical fields on MCSs are dose dependent. To evaluate the threshold of lethal effects (corresponding to irreversible membrane breakdown) of DC electrical fields, non-necrotic MCSs (150–350 μm in diameter) were treated for 60 s with different field strengths. No electrical field-induced cell lethality was observed below 2000 V $m^{-1}$ (Figure 1) ($n$ = 30 MCSs in each data point, three independent experiments, mean ± s.e.). Cell lethality increased when the field strength exceeded 3000 V $m^{-1}$ and reached a plateau at a field strength of $10^4$ V $m^{-1}$.

### Effects of DC electrical fields on membrane potential of MCSs

The changes in the membrane potential induced by superimposed external DC fields of different field strengths can be estimated according to Bernhard and Pauly (1973). A theoretical estimation of membrane potential change was performed for MCS of different diameters (Figure 2A). At a given external field strength

© Cancer Research Campaign 1997

**Figure 1** Effects of electrical fields with different field strengths on cell vitality in MCSs. After electrical field treatment MCSs were stained with Ethd-1 and the number of Ethd-1-positive cell nuclei were counted from the whole area under inspection the induced maximum membrane potential in MCSs is higher in large spheroids than small spheroids. At a critical membrane potential of 1 V, dielectric breakdown occurs in biological membranes (Weaver, 1993). Using the membrane potential-sensitive dye di-8-ANNEPS and the CLSM technique, membrane potential changes in MCSs during electrical field treatment were monitored. The application of an external DC electrical field pulse (field strength of 2000 V $m^{-1}$, duration of 6 s) resulted in a marked change in the relative fluorescence of di-8-ANNEPS (Figure 2B and C). During electrical field treatment, MCSs showed a depolarization at the cathode-facing side indicated by a drop in fluorescence, whereas at the anode-facing side a hyperpolarization (fluorescence increase) was observed ($n$ = 6). For a field strength of 2000 V $m^{-1}$ and a MCS diameter of 150 μm a membrane potential change of 225 mV was calculated.

### Expression of c-fos antigen

Activation of immediate-early response genes such as c-*fos*, c-*myc*, c-*jun*, *egr-1* as a result of various cellular stress stimuli have been reported in several studies (Sisken et al, 1993; Krukoff et al, 1994; Goldspink et al, 1995; Yoshida et al, 1995). As c-fos is a well-known mediator of cell proliferation, the expression of c-fos in MCSs after electrical field exposure was evaluated by immunohistochemistry. MCSs were treated with a single electrical field pulse of 500 V $m^{-1}$ for 60 s, which is below the threshold of membrane breakdown. Aliquots of the electropulsed MCSs (30, 60, 120, 240 and 480 min after DC field application) were fixed in ethanol–acetone and stained with an anti c-fos polyclonal antibody. c-fos antigen was significantly increased by a factor of 1.3 when evaluated 30 min after electrical field treatment. The c-fos level reached a maximum (increase by a factor of 2.6) at 120 min and was back-regulated towards the control level within 480 min (Figure 3A and B) ($n$ = 20 MCSs in each data point, three independent experiments, mean ± s.e.).

### Electrical field induced growth stimulation of MCS

When non-necrotic MCSs (300 ± 50 μm diameter) were treated with the same electrical field as used for c-fos induction (field strength of 500 V $m^{-1}$, duration of 60 s), an increased growth kinetics of MCS was observed (Figure 4). Evaluation of spheroid volumes over a time period of 6 days after the electropulse revealed that from day 2 treated MCSs were significantly larger ($P \leq 0.05$) than controls ($n$ = 20 MCSs in each data point, three independent experiments, mean ± s.e.). Whereas the volume-doubling time amounted to 4–5 days in control MCSs, it was reduced to 1–2 days in the electrical field-treated sample. The Gaussian distribution (Figure 5) of MCS diameters in the control sample before electrical field treatment and at day 6, as well as in the electropulsed sample at day 6, showed that the shape of the function remained unaltered. The function was, however, significantly ($P \leq 0.05$) shifted from 296 ± 61 μm (mean ± s.d., $n$ = 93) to larger spheroid diameters of 503 ± 52 μm (mean ± s.d., $n$ = 92) in the electrical field-treated sample. The MCS diameter in the control sample at day 6 amounted to 425 ± 51 μm (mean ± s.d., $n$ = 86).

### The necrotic core in electrical field-treated and control MCSs

Central necrosis of MCSs was examined using LYVS as a lethal stain. The diameter of the necrotic core was decreased in electrical field-treated samples when examined 6 days after application of a single electrical field pulse (500 V $m^{-1}$, 60 s) (Figure 6). The critical diameter for the first appearance of necrotic areas larger than 50 μm within MCSs was 350 ± 29 μm in control spheroids and 450 ± 25 μm in electrical field-treated spheroids ($n$ = 10 in each data point, three independent experiments, mean ± s.e.). A significant decrease ($P \leq 0.05$) in the necrosis diameter of electrical field-treated MCS was observed in all size classes of MCSs up to a diameter of 600 μm. Our data show that a decrease in necrosis diameter by 100 μm resulted in a 50-μm increase of the thickness of the vital rim of MCS.

### Thickness of the rim of proliferating cells in electrical field-treated MCSs

The vital cell rim of MCS consists of peripheral proliferating cells and more central layers of non-proliferating quiescent cells. The diameter of the proliferating rim of MCS 6 days after electrical field treatment was determined using the Ki-67 antibody. Ki-67 immunostaining was prominent in the peripheral cell layers whereas more central cell layers were predominantly Ki-67 negative, indicating quiescent cells. Interspersed with these quiescent cells single proliferating cells were found (Figure 7A). A threshold of $2 \times 10^3$ nuclei $mm^{-2}$ was set to distinguish between proliferating and quiescent cell layers. In a MCS size class of 400 ± 50 μm the rim of proliferating cells in electrical field-treated MCSs was significantly increased ($P \leq 0.05$) to 107 ± 11 μm ($n$ = 6, mean ± s.e.) as compared with control (60 ± 6 μm, $n$ = 6, mean ± s.e.) (Figure 7B). In control spheroids, about five cell layers of proliferating cells are located at the outermost periphery, whereas in electrical field-treated spheroids about ten cell layers contain proliferating cells. The thickness of the rim of quiescent cells was calculated by subtracting from the radius of the whole spheroid the radius of the necrosis and the thickness of the rim of proliferating cells. In a MCS size class of 400 ± 50 μm the thickness of the rim of quiescent cells was calculated to be 65 ± 10 μm ($n$ = 6, mean ± s.e.) and 73 ± 11 μm ($n$ = 6, mean ± s.e.) in control and electropulsed MCSs, respectively, which was not significantly

*© Cancer Research Campaign 1997* 

**Figure 2** Changes in membrane potential of MCS due to an external electrical field. (**A**) Theoretical estimation of the generated maximum membrane potential at the pole cap of MCSs (cos $\alpha = 1$) as a result of the application of different field strengths according to $V = 1.5 \times a \times E \times \cos \alpha$. E is the externally applied DC field, *a* is the spheroid radius and $\alpha$ represents the angle between a certain site of the MCS and the vector of the electrical field. Note that the membrane potential at a given external field strength depends on the spheroid size. The dotted horizontal line indicates the threshold for membrane breakdown. (**B**) Representative MCSs stained with di-8-ANNEPS during electrical field treatment with the cathode-facing side upward and the anode-facing side downward. The focus is in the equatorial plane of the MCSs (subtractive image, false colours, bar = 25 µm). (**C**) Time course of membrane potential during electrical field treatment. An electrical field of 2000 V m$^{-1}$ was applied during the period of time indicated by arrows. Depolarization at the cathode-facing side results in a drop of fluorescence, whereas at the anode a fluorescence increase was observed. Data are presented in arbitrary units as relative fluorescence variation $F/F_0$ with respect to the resting level $F_0$ (representative tracing)

different. An increase in the thickness of the rim of proliferating cells and a decrease in the necrosis diameter, however, shifts the rim of quiescent cells towards more central regions of the MCSs. The absolute number of quiescent cells was therefore reduced to 18% in electropulsed MCSs as compared with control, although the thickness of the rim of quiescent cells remained unaltered.

### DISCUSSION

In this study treatment of human prostate cancer spheroids with one single electrical field pulse led to a rapid and pronounced induction of c-fos, an enhanced MCS growth and an alteration of MCS vitality patterns. The enhanced growth was mediated via a reversal of cell quiescence to cell proliferation in deeper cell layers of MCSs. We have evaluated the conditions of electrical field treatment of MCSs leading to enhanced growth stimulation. We found that below a critical field strength of 500 V m$^{-1}$ applied for 60 s no effects were observed (data not shown). Above 2000 V m$^{-1}$ electrical field treatment resulted in increased cell lethality as a result of irreversible breakdown of cell membranes. The imposed electrical field under our experimental conditions led to a hyperpolarization at the anode-facing side of the spheroid due to a

 © Cancer Research Campaign 1997

**Figure 3** Immunostaining of c-*fos* expression after electrical field treatment (500 V, 60 s) of MCSs. (**A**) c-fos immunostaining of representative MCSs. Focus is on the surface of MCSs (bar = 100 µm). (**B**) Relative fluorescence increase (%) of c-fos cross-reactivity at different time points after electrical field treatment. Control (primary and secondary antibody) was set at 100%. ▨, Electrical field-treated MCSs; □, control

*© Cancer Research Campaign 1997*

**Figure 4** Electrical field-induced growth stimulation of MCSs. MCSs were treated with a single electrical field pulse (500 V, 60 s) and MCS volumes were recorded over 6 days. —●—, Electrical field-treated MCSs; —○—, control

**Figure 6** The necrotic core in electrical field treated (500 V, 60 s) (●) and control (□) MCSs. LYVS lethal staining 6 days after electrical field treatment. The diameter of the necrotic core is reduced in the electrical field-treated sample

**Figure 5** Gaussian distribution of control (□) and electrical field-treated (△) MCS. Diameters before (○) and 6 days after (△) electrical field (500 V, 60 s) treatment. Nonlinear curve fitting was performed according to the Gaussian algorithm. The Gaussian is shifted to the right in the electrical field-treated sample superimposition of the electrical field vector of the cell membrane potential and the electrical field vector of the external DC field, which are acting in the same direction. At the cathode-facing side a depolarization due to opposing external DC field and cell membrane potential vectors was observed. The primary event during electrical field treatment is the superimposition of the field vectors. The force of the external field induces ion movements and polarization processes in the vicinity of the cell membrane without changing the specific conductivity of the membrane. As a secondary effect accompanying the electrical field-induced membrane potential changes voltage-dependent ion fluxes, i.e. $Ca^{2+}$, $Na^{+}$, $K^{+}$, and $Cl^{-}$ channels may be activated.

The stimuli bringing non-proliferating quiescent cells within avascular MCS to proliferative activity are hardly known (Folkman, 1992). In the present study, evaluation of the vital rim by staining proliferating cells with the Ki-67 antibody showed an increased diameter of the rim of proliferating cells in electropulsed MCSs as compared with controls. Necrosis staining by LYVS of MCSs 6 days after electrical field treatment, revealed a decreased necrosis diameter in electropulsed MCS. Owing to the reduced necrosis diameter unstained quiescent cell layers were found in more central regions of MCSs, which lowered the absolute number of quiescent cells. These observations indicate that, following electrical field treatment, quiescent cells regain cell cycle activity and therefore the diameter of the rim of proliferating cells augments. Our data cannot be explained by an activation of quiescent cells due to electrical field-induced cell death in the peripheral proliferating cell layers as the field strength applied in our experiments was below the threshold of reversible or irreversible breakdown of cell membranes. This also excludes the possibility that growth factors or hormones from electropermeabilized peripheral cells were transferred into quiescent cell layers by electrophoretic processes. The external electrical field was, however, strong enough to alter the membrane potential. We therefore postulate that the cell membrane is the primary target for externally applied electrical fields. The change in the membrane potential could be the beginning of a signal transduction cascade leading to immediate early gene expression and subsequent cell cycle activation. We have previously shown (M Wartenberg, unpublished results) that a single electropulse (500 V $m^{-1}$, 60 s) led to an increase in intracellular reactive oxygen species and a transient

© Cancer Research Campaign 1997

**Figure 7** Ki-67 immunostaining of proliferating cells in control and electrical field treated (500 V, 60 s) MCSs. (**A**) Representative MCSs, cryosection. Left side, control. Right side, electrical field-treated sample (bar = 100 µm). (**B**) Number of Ki-67-positive nuclei mm$^{-2}$ from the periphery to the centre of the MCSs shown in (**A**). In the electrical field-treated sample (□) more central cell layers show positive Ki-67 staining than in the control (▨)

increase in intracellular $Ca^{2+}$, which are both well-known in activating immediate early-response genes and in promoting cell growth (Shibanuma et al, 1990; Nose et al, 1991; Werlen et al, 1993; Ohba et al, 1994; Roche and Prentki, 1994). The data of the present study indicate stimulation of immediate-early response genes as revealed by anti-c-fos immunohistochemistry. The c-*fos* immediate early gene is induced in various preparations by several stress factors, including light (Yoshida et al, 1995), photodynamic therapy (Kick et al, 1996), hyperosmolar solutions (Wollnik et al, 1993), ischaemia, hypoxia, mechanical stimuli (Goldspink et al, 1995) and electrical nerve stimulation (Krukoff et al, 1994). Additionally, transient transcription of the c-*fos* gene has been reported during serum stimulation of quiescent cells in monolayer culture (Pai and Bird, 1994). The data of the present study suggest that a single short-lasting electrical-field pulse enhances tumour growth by activating quiescent cells in the depth of the tissue. The stimulatory effect of electrical fields on reversal of cancer cell dormancy should be kept in mind when patients are treated by electrotherapeutic approaches. It should, however, also be considered that a targeted activation of the highly therapy-resistant quiescent cell layers, i.e. by controlled electrical-field exposure, may be useful to sensitize tumours to radiation and chemotherapy.

### REFERENCES

Acker H. (1984). Microenvironmental conditions in multicellular spheroids grown under liquid-overlay tissue culture conditions. In *Spheroids in Cancer Research*, Acker H, Carlsson J, Durand, RE and Sutherland RM. (eds.) vol. **95**, pp. 116–133. Springer-Verlag, Berlin.

Azavedo E, Svane G and Nordenström B (1991) Radiological evidence of response to electrochemical treatment of breast cancer. *Clin Radiol* **43**: 84–87

Belehradek M, Domenge C, Luboinski B, Orlowski S, Belehradek J, JR and Mir LM (1993) Electrochemotherapy, a new antitumor treatment. First clinical phase I–II trial. *Cancer* **72**: 3694–3700

Bernhardt J and Pauly H (1973) On the generation of potential differences across the membrane of ellipsoidal cells in an alternating electrical field. *Biophysik* **10**: 89–98

Carlsson J, Nilsson K, Westermark B, Pontén J, Sundström C, Larsson E, Bergh J, Pahlman S, Busch C. and Collins VP (1983) Formation and growth of multicellular spheroids of human origin. *Int J Cancer* **31**: 523–533

Carlsson J, Daniel-Szolgay E, Frykholm G, Glimelius B, Hedin A and Larsson B (1989) Homogeneous penetration but heterogeneous binding of antibodies to carcinoembryonic antigen in human colon carcinoma HT-29 spheroids. *Cancer Immunol Immunother* **30**: 269–276

Folkman J. (1992). The role of angiogenesis in tumor growth. *Semin Cancer Biol* **3**: 65–71

Goldspink DF, Cox VM, Smith SK, Eaves LA, Osbaldeston NJ, Lee DM and Mantle D (1995) Muscle growth in response to mechanical stimuli. *Am J Physiol* **268**: E288–E297

Gross D, Loew LM, Webb WW (1986) Optical imaging of cell membrane potential changes induced by applied electric fields. *Biophys J* **50**: 339–348

Kick G, Messer G, Plewig G, Kind P and Goetz AE (1996) Strong and prolonged induction of c-*jun* and c-*fos* proto-oncogenes by photodynamic therapy. *Br J Cancer* **74**: 30–36

Kloth LC and Feedar JA (1988) Acceleration of wound healing with hight voltage, monophasic, pulsed current. *Phys Ther* **68**: 503–508

Krukoff TL, Harris KH, Linetsky E and Jhamandas JH (1994) Expression of c-fos protein in rat brain elicited by electrical and chemical stimulation of the hypothalamic paraventricular nucleus. *Neuroendocrinology* **59**: 590–602

Nose K, Shibanuma M, Kikuchi K, Kageyama H, Sakiyama S, and Kuroki, T (1991) Transcriptional activation of early-response genes by hydrogen peroxide in a mouse osteoblastic cell line. *Eur J Biochem* **201**: 99–106

Ohba M, Shibanuma M, Kuroki, T and Nose K (1994) Production of hydrogen peroxide by transforming growth factor-beta 1 and its involvement in induction of egr-1 in mouse osteoblastic cells. *J Cell Biol* **126**: 1079–1088

Pai SR and Bird RC (1994) c-fos expression is required during all phases of the cell cycle during exponential cell proliferation. *Anticancer Res* **14**: 985–994

Roche E and Prentki M (1994) Calcium regulation of immediate-early response genes. *Cell Calcium* **16**: 331–338

Sersa G, Cemazar M and Miklavcic D (1995) Antitumor effectiveness of electrochemotherapy with *cis*-diamminedichloroplatinum (II) in mice. *Cancer Res* **55**: 3450–3455

Shibanuma M, Kuroki T and Nose K (1990) Stimulation by hydrogen peroxide of DNA synthesis, competence family gene expression and phosphorylation in quiescent Balb/3T3 cells. *Oncogene* **3**: 27–32

Singh S and Katz JL (1986) Scientific basis of electro-stimulation. *J Bioelec* **5**: 285–327

Sisken BF, Walker J and Orgel M (1993) Prospects on clinical applications of electrical stimulation for nerve regeneration. *J. Cell. Biochem*, **52**: 404–409

Soranzo C and Ingrosso A (1988) A comparative study of the effects of anthracycline derivatives on a human adenocarcinoma cell line (LoVo) grown as a monolayer and as spheroids. *Anticancer Res* **8**: 369–374

Stuschke M, Budach V and Sack H (1993) Radioresponsiveness of human glioma, sarcoma, and breast cancer spheroids depends on tumor differentiation. *Int J Radiation Oncol Biol Phys* **27**: 627–636

Sutherland RM, McCredie JA and Inch WR (1971) Growth of multicell spheroids in tissue culture as a model of nodular carcinomas. *J Natl Cancer Inst* **46**: 113–117

Sutherland RM (1988) Cell and environment interactions in tumor microregions: the multicell spheroid model. *Science* **240**: 177–184

Wartenberg M and Acker H (1995) Quantitative recording of vitality patterns in living multicellular spheroids by confocal microscopy. *Micron* **26**: 395–404

Wartenberg M. and Acker H (1996) Induction of cell death by doxorubicin in multicellular spheroid as studied by confocal laser scanning microscopy. *Anticancer Res* **16**: 573–580

Weaver JC (1993) Electroporation: a general phenomenon for manipulating cells and tissues. *J Cell Biochem* **51**: 426–435

Werlen G, Belin D, Conne B, Roche E, Lew D, and Prentki M (1993) Intracellular $Ca^{2+}$ and the regulation of early response gene expression in HL-60 myeloid leukemia cells. *J Biol Chem* **268**: 16596–16601.

Wollnik B, Kubisch C, Maass A, Vetter H and Neyses L (1993) Hyperosmotic stress induces immediate-early gene expression in ventricular adult cardiomyocytes. *Biochem Biophys Res Commun* **194**: 642–646

Yoshida K, Imaki J, Masuda H, Hagiwara M (1995) Light-induced CREB phosphorylation and gene expression in rat retinal cells. *J Neurochem* **65**: 1499–1504

 © Cancer Research Campaign 1997